# Adverse pregnancy outcomes and subsequent risk of cardiovascular disease in women with systemic lupus erythematosus

**DOI:** 10.1186/ar4662

**Published:** 2014-09-18

**Authors:** Pin Lin, Elisa Rhew, Roberta B Ness, Alan Peaceman, Alan Dyer, David McPherson, George T Kondos, Daniel Edmundowicz, Kim Sutton-Tyrrell, Trina Thompson, Rosalind Ramsey-Goldman

**Affiliations:** 1 Northwestern University, Feinberg School of Medicine, Chicago, IL, USA; 2 The University of Texas, School of Public Health, Houston, TX, USA; 3 University of Texas Health Science Center, Houston, TX, USA; 4 University of Illinois, Chicago, IL, USA; 5 Temple University School of Medicine, Philadelphia, PA, USA; 6 University of Pittsburgh, Graduate School of Public Health, Pittsburgh, PA, USA

## Background

Patients with systemic lupus erythematosus (SLE) are at increased risk for adverse pregnancy outcomes and cardiovascular disease (CVD). The objective of this exploratory study was to investigate the association between a history of adverse pregnancy outcomes and subsequent risk of subclinical CVD assessed by imaging studies and verified clinical CVD events in 129 women with SLE.

## Methods

The occurrence of adverse pregnancy outcomes, specifically pre-eclampsia, preterm birth, and low birth weight, was ascertained by questionnaire. Subclinical CVD was assessed by coronary artery calcium (CAC) as measured by electron beam computed tomography (EBCT) and carotid plaque measured by B-mode ultrasound. Clinical CVD events were verified by medical record review. Logistic regression was used to estimate the association of pregnancy complications with occurrence of subclinical and clinical CVD with *a priori *adjustment for age, which is associated with CVD and SLE disease duration as a measure of SLE disease burden.

## Results

Fifty-six women reported at least one pregnancy complication while 73 had none. Twenty-six women had at least one pregnancy complicated by pre-eclampsia and were more likely to have a CAC score ≥10 (adjusted odds ratio (OR) = 3.7; 95% confidence interval (CI): 1.2, 11.9), but the presence of plaque was not associated with this pregnancy complication (OR = 1.1; 95% CI: 0.4, 2.8). Low birth weight and preterm birth were not associated with CAC or plaque.

## Conclusions

SLE patients with a history of pre-eclampsia had a higher rate of subclinical CVD as measured by CAC score. Future studies are needed to confirm the relationship between adverse pregnancy outcomes and subsequent subclinical CVD and clinical CVD events.

